# Ataxia as the main manifestation of tumor-like primary angiitis of the central nervous system: a case report and literature review

**DOI:** 10.1186/s12880-019-0382-9

**Published:** 2019-10-15

**Authors:** Yu-Jing Huang, Li Zhang, Ying Mao, Guang-Xian Nan

**Affiliations:** Department of Neurology, China-Japan Union Hospital of Jilin University, No.126 Xiantai Street, Changchun, 130033 Jilin China

**Keywords:** Ataxia, Tumor-like primary angiitis of the central nervous system, Brain MRI examination, Glucocorticoid therapy

## Abstract

**Background:**

Primary angiitis of the central nervous system (PACNS) is a rare disease, and tumor-like primary angiitis of the central nervous system is even rarer. Histopathology is the gold standard for tumor-mimicking PACNS. However, pathological diagnosis is relatively limited due to fewer biopsy opportunities.

**Case presentation:**

A 68-year-old male presented with ataxia, and was diagnosed with tumor-like primary angiitis of the central nervous system. The patient underwent Intravenous drip glucocorticoid therapy (10 mg of dexamethasone, daily). After 10 days, the symptoms of the patient were completely relieved. Radiology revealed that the low density lesion in the right cerebellar hemisphere obviously narrowed. Cyclophosphamide therapy was not initiated.

**Conclusion:**

It is crucial for clinicians to be aware of changes in radiology that indicate PACNS, since the diagnosis of tumor-like PACNS remains quite challenging. Glucocorticoid therapy is an effective therapy in this condition, and the prognosis can be favorable.

## Background

Primary angiitis of the central nervous system (PACNS) is a rare disease. The etiology of this disease remains unknown, and it has no correlation with infection and autoimmunity. PACNS mainly affects the small or medium vessels (arteries or veins), and the leptomeninges of the brain and spinal cord. A subtype of PACNS is called tumor-like PACNS, in which the imaging features a solitary tumor-like mass lesion. In order to improve the early diagnosis and identification of tumor-mimicking PACNS, one case of tumor-like PACNS in our hospital, in which ataxia was the main manifestation, was reported.

## Case presentation

A 68-year-old male was admitted to our hospital due to dizziness and unstable walking. The patient also complained of headache, which persisted for the whole duration of the disease. Furthermore, the patient had a history of hypertension and diabetes, traumatic splenectomy, and gout.

The physical examination revealed a blood pressure (BP) of 180/90 mmHg (normal range: 80–120/60–80 mmHg). Furthermore, the patient’s right eyeball abduction was restricted, the right finger test was less stable, and the right side and knee tibia test were less stable.

The laboratory investigations indicated a white blood cell (WBC) count of 12.56 × 10^9^/L (reference range: 4–10 × 10^9^ cells/L), a lymphocyte count of 4.86 × 10^9^ cells/L (0.8–4.0 × 10^9^ cells/L), and a monocyte count of 0.92 × 10^9^ cells/L (0.12–0.80 × 109 cells/L). Furthermore, blood sugar was 9.53 mmol/L, urine glucose was 2+, triglyceride was 7.2 mmol/L (< 1.7 mmol/L), and total cholesterol was 7.18 mmol/L (3.00–5.70 mmol/L).

Lumbar puncture and cerebrospinal fluid (CSF) analyses were performed. CSF pressure was 130 mmH_2_O, CSF WBC count was 6 × 10^9^ cells/L (0–8 × 10^9^ cells/L), CSF glucose was 3.2 mmol/L (2.2–3.9 mmol/L), CSF protein was 0.2 g/L (0.12–0.60 g/L), and CSF chloride was 127.2 mmol/L (120–132 mmol/L).

**Fig. 1:**
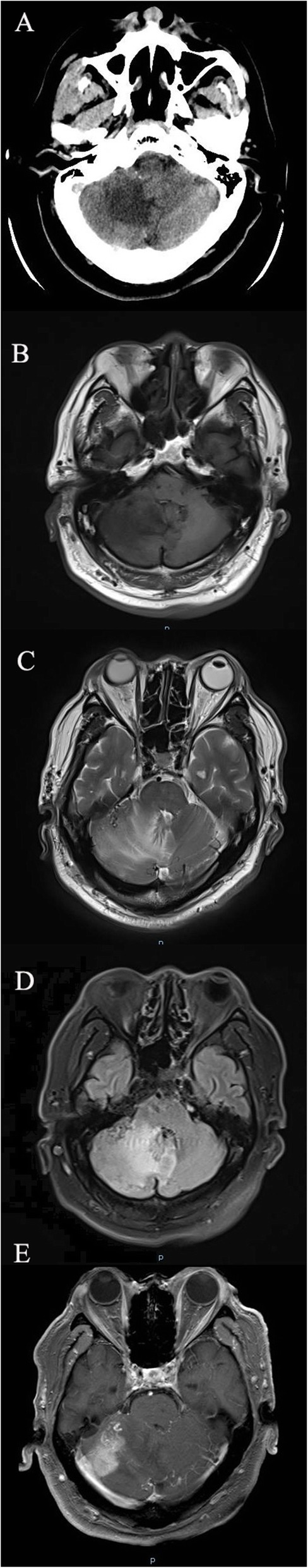
**a**. The brain CT scan revealed a large area of low-density lesion in the right cerebellar hemisphere. **b**. The brain MRI T1 weighted image revealed a large area of long T1 signal in the right cerebellar hemisphere with an obvious space-occupying effect. The brain stem and fourth ventricle were obviously pressed, and moved slightly to the left. **c**. The brain MRI T2 weighted image revealed a large area of long T2 signal in the right cerebellar hemisphere, with an obvious space-occupying effect. The brain stem and fourth ventricle were obviously pressed, and moved slightly to the left. **d**. The brain MRI FLAIR weighted image revealed a large area of hyperintense signal in the right cerebellar hemisphere, with an obvious space-occupying effect. The brain stem and fourth ventricle were obviously pressed, and moved slightly to the left. **e**. The brain MRI T1 weighted image enhanced sequence revealed that the lesions in the right cerebellar hemisphere were markedly enhanced by contrast media

**Fig. 2:**
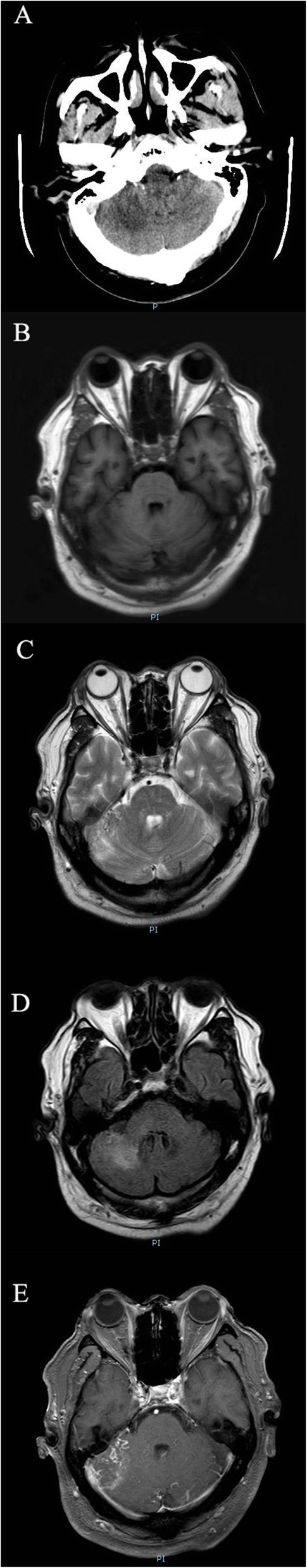
**a**. The brain CT scan after 10 days of treatment revealed that low-density lesions in the right cerebellar hemisphere were attenuated. **b**. The brain MRI T1 weighted image after 10 days revealed a less long T1 signal in the right cerebellar hemisphere. **c**. The brain MRI T2 weighted image after 10 days revealed that a long T2 signal in the right cerebellar hemisphere attenuated. **d**. The brain MRI FLAIR weighted image after 10 days revealed that a hyper-intense signal in the right cerebellar hemisphere attenuated. **e**. The brain MRI T1 weighted image enhanced sequence revealed that there were less lesions in the right cerebellar hemisphere

**Fig. 3:**
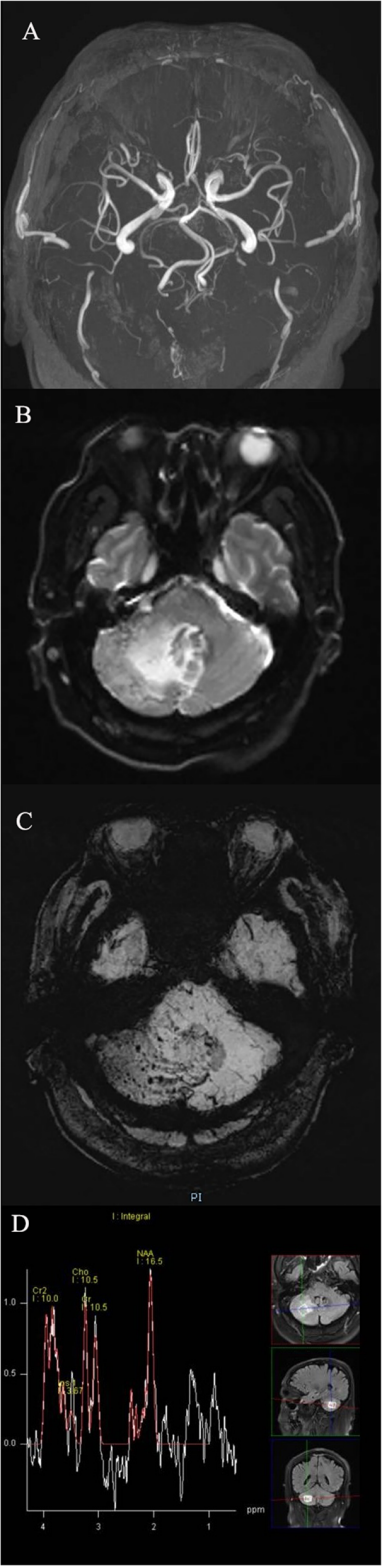
**a**. There was no abnormality in the magnetic resonance angiography. **b**. The diffusion weighted image revealed a large area of low-density lesion in the right cerebellar hemisphere. **c**. The susceptibility weighted imaging revealed multiple punctate micro-bleeding lesions in the right cerebellar hemisphere. **d**. The magnetic resonance spectroscopy revealed that the N-acetyl aspartate (NAA) peak and creatinine (Cr) had no significant changes, the choline (Cho) peak increased, NAA/Cr = 1.05, Cho/Cr = 1.53, and Cho/NAA = 0.95

The brain computed tomography (CT) result revealed a low density lesion in the right cerebellar hemisphere. The 3.0 T magnetic resonance imaging (MRI) was used to obtain the T1-weighted image, T2-weighted image, fluid-attenuated inversion recovery (FLAIR), diffusion weighted image (DWI), susceptibility weighted imaging (SWI), magnetic resonance angiography (MRA), magnetic resonance spectroscopy (MRS), and T1-weighted image enhanced sequence. The brain MRI suggested that the lesion had long T1 and T2 signals, and a high signal in the FLAIR image. The brain stem and fourth ventricle were obviously pressed, and moved slightly to the left. The DWI was not abnormal, and the MRA did not reveal any apparent anomalies. The SWI revealed a marked low signal on the lesion, the boundary was clear, and the phase map had a high and low mixed signal, which indicated a micro hemorrhage. The MRI enhancement in the brain revealed the lateral marginal margin of the right cerebellar hemisphere, in which the obvious enhancement area of the plaque was visible, the local cerebellar gyrus was clear, and the right cerebellar hemisphere was visible with a strip and tortuous vascular shadow. No obvious inflammatory lesions were found in the chest CT. The MRS revealed a right cerebellar hemisphere lesion (PRESS, TR = 2000 ms, TE = 30 ms, 135 ms). Furthermore, the N-acetyl aspartate (NAA) peak and creatinine (Cr) had no significant change, the choline (Cho) peak increased, NAA/Cr = 1.05, Cho/Cr = 1.53, and Cho/NAA = 0.95 (Figs. 1, 2, 3).

Additional tests were performed to exclude any underlying systemic disease or alternative diagnoses (− for negative, + for positive):

1. **Systemic rheumatic diseases:**
  Serum Antinuclear antibodies (ANA), 1:100 (−);
  dsDNA antibodies, (−);
  Antiphospholipid antibodies-Lupus anticoagulant (LA), (−);
  Anticardiolipin (aCL) antibodie, (−);
  Anti-beta2 glycoprotein (GP) antibodies, (−);
  Antineutrophil cytoplasmic antibodies (ANCA), (−);
  Serum C3: 0.96 g/L, (normal range: 0.9–1.5 g/L);
  Serum C4 0.24 g/L, (0.2–0.4 g/L);
  IgG, 9.3 g/L (8–16 g/L); IgM, 1.13 g/L (0.5–2.2 g/L); IgA, 1.99 g/L (8–16 g/L).
2. **Infectious diseases:**
  Syphilis: Venereal disease research laboratory (VDRL), (−);
  Rapid plasma reagin (RPR), (−); *T. pallidum* particle agglutination assay (TPPA), (−);
  Tuberculosis (TB): Acid-fast bacilli smear and mycobacterial culture, (−); Chest CT for pulmonary TB infection, (−); TB-PCR test, (−);
  Hepatitis B and C viruses: Serum antigen and antibody tests, (−);
  Human Immunodeficiency Virus (HIV): Serum antibody test, (−).

Since no alternative diagnosis was achieved, according to the results of the brain MRI scan and functional imaging, the suspected diagnosis was PACNS, and an intravenous infusion of 10 mg of dexamethasone per-day was given for the consecutive 10 days. After steroid therapy, the symptoms of the patient were completely relieved. The brain CT revealed that the low density lesion of the right cerebellar hemisphere obviously narrowed, and the standard and enhanced MRI scan were re-checked. The plaques were long T1 and T2 signals, the FLAIR was slightly high, and the enhanced scan revealed a flaky enhancement. Thus, cyclophosphamide therapy was not initiated.

## Discussion

The first symptom of the present case was ataxia. Even though the blood lymphocyte and monocyte counts were slightly elevated, none of the symptoms or tests (including the CSF analysis) indicated infection. Furthermore, the results of the laboratory examination did not support syphilis or other infections, leukemia, antiphospholipid antibody syndrome, or other rheumatic disease that may cause vasculitis. In addition, disease-like chronic lymphocytic inflammation with pontine perivascular enhancement responsive to steroids (CLIPPERS) were excluded for the non-conformity of the MRI (the lesion of the CLIPPERS scattered throughout the pons with variable involvement of the medulla, brachium pontis, cerebellum, midbrain and spinal cord). Combined with imaging examination, the preliminary clinical diagnosis of PACNS can be made, since no secondary vasculitis or alternative diagnoses was present. Although the patient could not cooperate with the pathological biopsy, the patient’s symptoms improved after steroid therapy, and the imaging of the lesions significantly improved, which further supported the PACNS diagnosis.

PACNS was proposed for the first time as an independent disease by Cravioto et al. in 1959 [1]. In 2008, a new subtype of tumor-like primary angiitis of the central nervous system [2] was proposed by Molloy et al. after summarizing the information of 38 patients with tumor-like lesions and the pathological examination of PACNS.

## Clinical manifestation

The incidence of PACNS is 2.4/100 per year, and lump-like PACNS accounts for 5–15% [3] of PACNS. This can occur at any age, with no gender preference. Furthermore, the clinical manifestations are correlated to the size of the involved vessel and the pathological type of vasculitis. Headache, cognitive impairment and persistent focal neurological deficits or stroke are the most common clinical manifestations of PACNS [4]. Headache is the first symptom for a mass-like tumor.

## Laboratory examination

The laboratory examination was not specific and sensitive, the erythrocyte sedimentation rate was 2–3 times higher than the normal value, and the serum autoantibodies were mostly negative [5]. Cytosolic ANCA and ANCA were weak positive [6] in some patients. Furthermore, 90% of the results of the cerebrospinal fluid of PACNS patients were abnormal, and the protein content and number of WBC could be mild to moderate.

## Imaging features

### Brain CT examination

One-third to two-thirds of PACNS patients can present different degrees of abnormal low density signals, and its sensitivity is lower than MRI.

### Brain MRI examination

MRI T1 weighted images, T2 weighted images and fluid attenuated inversion recovery sequences are highly sensitive to lesions, but lack specificity. PACNS is mainly observed in unilateral supratentorial lesions (frontal lobe, temporal lobe, occipital lobe and parietal lobe), with the frontal lobe as the center.

MRI enhanced scans can monitor blood flow changes, and the thickening and intensifying of the blood vessel wall at the early stage. The characteristics of focus enhancement are more common in unilateral supratentorial cerebral hemispherical mass enhancement, which can be reinforced with banded and linear, and are partly accompanied by edema and an occupying effect, and the midline displacement is also visible.

DWI usually presents with a high signal, and the apparent diffusion coefficient has a low signal. It is difficult to identify some diseases simply with DWI. In practice, it is necessary to comprehend the characteristics of the change in DWI signal as disease evolving, and improve its value in the diagnosis of lump-like PACNS.

MRS has important clinical significance for the evaluation of lesions, such as PACNS. The Cho/Cr, Cho/NAA, lipid and lactic acid peaks were all increasing. It has also been reported that the peak value of glutamate and glutamine in the MRS of PACNS patients is significantly higher. Panchal et al. [7] further inferred that glutamine synthetase is activated under inflammatory reactions, and that the glutamic acid produced by glutamic acid is increased.

SWI is important in diagnosing PACNS. Its magnetic susceptibility differential imaging based on blood oxygen saturation has great advantages in hemorrhage and small vessel imaging. Since the blood vessels of PACNS patients were liable to rupture and hemorrhage, SWI would be helpful for its diagnosis and differential diagnosis.

MRI perfusion imaging is a non-invasive method that reflects the distribution and perfusion of tissue microcirculation. Tumor-mimicking PACNS can lead to a decrease in local blood flow. The study conducted by Muccio [8] revealed that the PWI of PACNS has low perfusion, while the PWI of gliomas has high perfusion. Therefore, PWI has great significance in the identification of lesions, such as PACNS and brain tumors.

### Digital subtraction angiography

Few abnormal lesions were found. The typical changes included uneven thickness of the vessels, segmental stenosis, dilatation, vascular obstruction and delayed vascular emptying. However, there was a lack of specificity. Furthermore, DSA cannot be used as a routine examination for lump-like PACNS [9].

## Brain tissue biopsy

Histopathological examination is the gold standard for the diagnosis of PACNS, but the positive rate of brain biopsy is only 25–50% [10]. PACNS histopathological results can be divided into four types: granulomatous vasculitis, lymphocytic vasculitis, necrotic vasculitis, and beta amyloid-associated cerebral vasculitis (A beta-related angitis, A beta RA) [11].

## Diagnosis

Calabrese and Mallek proposed the diagnostic criteria in 1988 [12]. At present, this standard is still in use. The details are as follows: (1) Clinical criteria. The patient’s history or clinical examination suggests that there are neurological deficits. (2) The criteria for imaging and histology. The vasculitis of the central nervous system is confirmed by imaging and/or pathology. (3) Exclusion criteria. There is no evidence of systemic vasculitis, or any evidence that the vasculitis is secondary, such as syphilis associated vasculitis. All of the above conditions should be met.

## Treatment and prognosis

The treatment of PACNS has not been supported by randomized controlled trials. The recommended treatment is glucocorticoid combined with immunosuppressive agents [13]. However, there have been reports that the efficacy of a steroid alone is better [14]. Usually, steroid therapy is initiated with 1 mg/kg of prednisone to a maximum of 80 mg/day, or its equivalent. The dosage of steroids should be tapered after four to 6 weeks of initial dosage, or according to the finally reached dosage of 5 mg of prednisone, daily. For the present patient, after being suspected and diagnosed with PACNS, steroid alone was started, and the patient’s symptom and radiology tests were closely monitored. After 10 days, the symptoms of the patient were completely relieved, and lesions on MRI scan were alleviated. Thus, cyclophosphamide therapy was not started.

## Conclusion

When a patient has central nervous system symptoms and the imaging findings are mass–like, but are not complete, tumor-like PACNS should be taken into consideration, and the laboratory and brain MRI examinations and functional imaging should be further examined. It is crucial for clinicians to be aware of the changes in radiology, which indicate PACNS, since the diagnosis of tumor-like PACNS is quite challenging. Glucocorticoid therapy is an effective therapy for this condition, and the prognosis can be favorable.

## Data Availability

Not applicable.

## Abbreviations

BP: Blood pressure
DWI: Diffusion weighted image
FLAIR: Fluid attenuated inversion recovery
MRA: Magnetic resonance angiography
MRS: Magnetic resonance spectroscopy
NAA: N-Acetyl aspartate
PACNS: Primary angiitis of the central nervous system
SWI: Susceptibility weighted imaging
WBC: White blood cell
